# Novel *DLX3* variants in amelogenesis imperfecta with attenuated tricho‐dento‐osseous syndrome

**DOI:** 10.1111/odi.12955

**Published:** 2018-09-09

**Authors:** Laura L. E. Whitehouse, Claire E. L. Smith, James A. Poulter, Catriona J. Brown, Anesha Patel, Teresa Lamb, Lucy R. Brown, Elizabeth A. O’Sullivan, Rowena E. Mitchell, Ian R. Berry, Ruth Charlton, Chris F. Inglehearn, Alan J. Mighell

**Affiliations:** ^1^ School of Dentistry University of Leeds Leeds UK; ^2^ Section of Ophthalmology and Neuroscience, Leeds Institute of Biomedical and Clinical Sciences University of Leeds Leeds UK; ^3^ Section of Genetics University of Leeds Leeds UK; ^4^ Birmingham Dental Hospital and School of Dentistry Birmingham UK; ^5^ Oxford University Hospitals NHS Foundation Trust Oxford UK; ^6^ City Health Care Partnership (CIC) Hull UK; ^7^ Leeds Genetics Laboratory St James’s University Hospital Leeds UK

**Keywords:** amelogenesis imperfecta, DLX3, enamel, trico‐dento‐osseous syndrome

## Abstract

**Objectives:**

Variants in DLX3 cause tricho‐dento‐osseous syndrome (TDO, MIM #190320), a systemic condition with hair, nail and bony changes, taurodontism and amelogenesis imperfecta (AI), inherited in an autosomal dominant fashion. Different variants found within this gene are associated with different phenotypic presentations. To date, six different *DLX3* variants have been reported in TDO. The aim of this paper was to explore and discuss three recently uncovered new variants in *DLX3*.

**Subjects and Methods:**

Whole‐exome sequencing identified a new *DLX3* variant in one family, recruited as part of an ongoing study of genetic variants associated with AI. Targeted clinical exome sequencing of two further families revealed another new variant of *DLX3* and complete heterozygous deletion of *DLX3*. For all three families, the phenotypes were shown to consist of AI and taurodontism, together with other attenuated features of TDO.

**Results:**

c.574delG p.(E192Rfs*66), c.476G>T (p.R159L) and a heterozygous deletion of the entire *DLX3* coding region were identified in our families.

**Conclusion:**

These previously unreported variants add to the growing literature surrounding AI, allowing for more accurate genetic testing and better understanding of the associated clinical consequences.

## 1 INTRODUCTION

Amelogenesis is the production of dental enamel, the hardest structure produced by the human body. The process is comprised of two major stages: the secretion and maturation stages. These result in an acellular, high‐strength hydroxyapatite structure with a distinct latticelike organization, designed to maintain function over a lifetime without the need for cellular repair (Robinson et al., 2000).

Amelogenesis imperfecta (AI) is a group of heterogeneous genetic conditions characterized by abnormal or absent enamel production. AI arises due to genetic changes affecting the secretory and/or maturation stages. Unlike many environmental causes of abnormal enamel, AI affects both the primary and secondary dentition. The severity of the condition is highly variable. AI can be divided into two major groups: hypoplastic (due to inadequate enamel volume) or hypomineralized (due to inadequate mineralization). However, mixed presentations exist and there is a spectrum of phenotypes. The prevalence of AI is reported to be between 1/700 and 1/14,000, depending upon the population studied (Bäckman & Holm, 1986; Witkop & Sauk, 1976). X‐linked, autosomal dominant and autosomal recessive inheritance patterns have all been reported for AI (Lacruz, Habelitz, Wright, & Paine, 2017; Smith et al., 2017).

AI may also be part of syndromic conditions such as tricho‐dento‐osseous syndrome (TDO) (MIM #190320, Jorgenson & Warson, 1973). TDO is a rare, autosomal dominant systemic genetic disease resulting from heterozygous mutations in *DLX3* (distal‐less homeobox 3) and has an unknown incidence rate. The majority of published cases were identified in the United States, specifically in Virginia, Tennessee and North Carolina (Price, Bowden, Wright, Pettenati, & Hart, 1998). All of the individuals reported to date presented with taurodontism and enamel hypoplasia. Robinson and Miller (1966) reported that the hair of affected individuals is classically coarse, kinky/curly, brittle and rough, with profuse shedding. This usually normalizes with age. Skeletal abnormalities within the head and neck have also been associated with TDO, including a lack of mastoid pneumatization, increased thickness of the cranial bones, increased bone density and a shortened mandibular ramus (Crawford & Aldred, 1990). The thickening of the bone in TDO may be of concern with regard to the increased risk of fracture or macrocephaly (Al‐Batayneh, 2012; Shapiro, Quattromani, Jorgenson, Young, & Opitz, 1983). However, Hart et al. (1997) stated that the bone changes are not associated with any pathology. Nails have also been reported to show splitting and to break easily (Quattromani et al., 1983; Wright, Kula, Hall, Simmons, & Hart, 1997). Atopic dermatitis was also noted in one individual with TDO (Mayer, Baal, Litschauer‐Poursadrollah, Hemmer, & Jarisch, 2010).

The clinical abnormalities associated with TDO are usually identified within the first year of life. Differentiating between TDO and amelogenesis imperfecta of the hypomaturation‐hypoplasia type with taurodontism (MIM #104510) can be difficult and is typically based upon other syndromic findings, such as kinky hair and bony changes. Both conditions have been associated with *DLX3* mutations suggesting the two conditions may be part of the same syndromic spectrum (Dong et al., 2005). However, Price et al. (1999) and Crawford and Aldred (1990) considered the two syndromes different entities. To address the controversy diagnosing TDO, Seow (1993) created criteria comprising generalized enamel defects; severe taurodontism affecting the mandibular first permanent molars; autosomal dominant inheritance; and at least one other feature (i.e., nail changes; bone sclerosis; or curly, kinky or wavy hair at a young age). However, because of the known clinical variations in the phenotypes of TDO, these criteria are not widely used.

The first mutation identified in TDO was a 4‐bp deletion (Price et al., 1998), leading to a frameshift variant (c.571_574delGGGG, p.(G190delfs*66)). Since the original report by Price et al. (1998), five other mutations have been described within *DLX3* (Dong et al., 2005; Harbuz et al., 2013; Li et al., 2015; Mayer et al., 2010; Nieminen et al., 2011) leading to attenuated versions of the disease (Wright et al., 2008).

This report presents two novel *DLX3* variants and heterozygous deletion of *DLX3* associated with AI. All families presented with AI prior to genetic testing. Subsequent to the identification of a *DLX3* variant in each family, affected individuals were reassessed clinically and were considered to have attenuated versions of TDO. We also report analysis of the hair phenotypes in family 1 and discuss the likely consequences of these newly identified mutations on the function of the DLX3 protein.

## 2 MATERIALS AND METHODS

### 2.1 Patients

**Figure 1:**
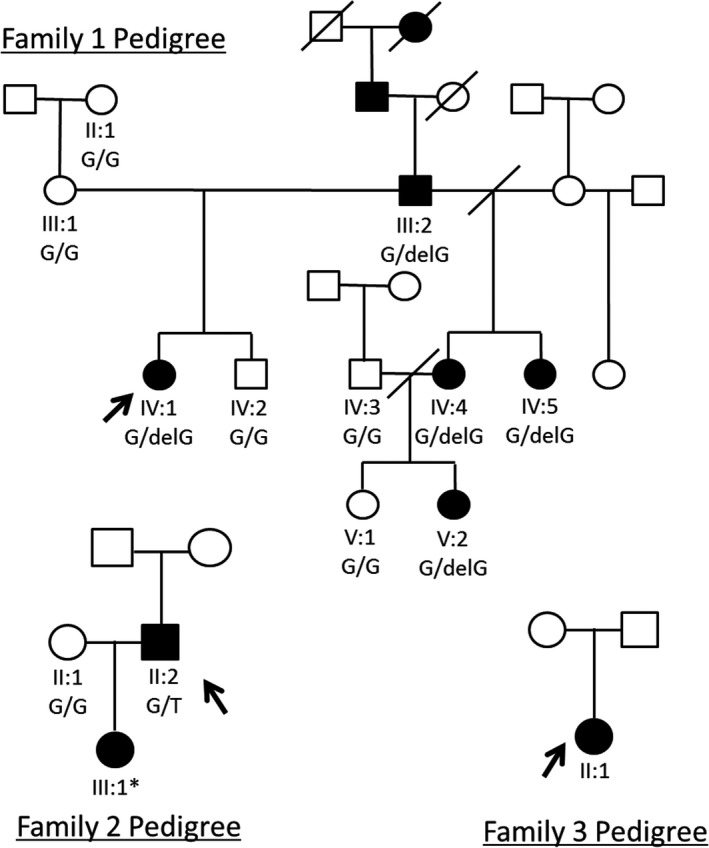
Pedigrees for families 1, 2 and 3. Probands whole‐exome sequenced/targeted clinical‐exome sequenced marked with arrows. * indicates that the DNA was of insufficient quality and quantity to sequence. ●: Affected female, □: Unaffected male

Eleven individuals from family 1, three individuals from family 2 and one individual from family 3 (Figure 1) were recruited following informed consent in accordance with the principles outlined in the Declaration of Helsinki and with local ethical approval by the National Research Ethics Service (South Yorkshire Research Ethics Committee ref: 13/YH/0028). For family 1, genomic DNA was obtained from saliva using Oragene® DNA Sample Collection kits (DNA Genotek, ON, Canada) according to the manufacturer's instructions. For families 2 and 3, genomic DNA was extracted from blood with the exception of individual III:1 (family 2), who deposited a buccal swab sample. Families 2 and 3 were screened as part of the recently developed NHS testing service for AI (available through the NHS UK Genetic Testing Network, https://ukgtn.nhs.uk/find-a-test/search-by-disorder-gene/details/6746/).

### 2.2 Whole‐exome and targeted clinical exome sequencing and analysis

Genomic DNA from individual IV:1 from family 1 (marked with an arrow on the pedigree, Figure 1) was subjected to whole‐exome sequencing (WES). Three micrograms of genomic DNA was processed using the SureSelect XT Library Prep and with the SureSelect Human All Exon v5 capture reagent according to the manufacturer's protocol (Agilent Technologies, Santa Clara, CA, USA). Sequencing was performed on an Illumina HiSeq 2500 (Illumina, San Diego, CA, USA) using a 100‐bp paired‐end protocol. The fastq files were aligned to the human reference genome (GRCh37) using the Burrows Wheeler aligner (BWA) (Li & Durbin, 2009). The resulting alignment was processed in the SAM/BAM format using the SAMtools, Picard (https://broadinstitute.github.io/picard/) and the Genome Analysis Toolkit (GATK) in order to correct alignments around indel sites and mark potential PCR duplicates (DePristo et al., 2011; McKenna et al., 2010).

**Figure 2:**
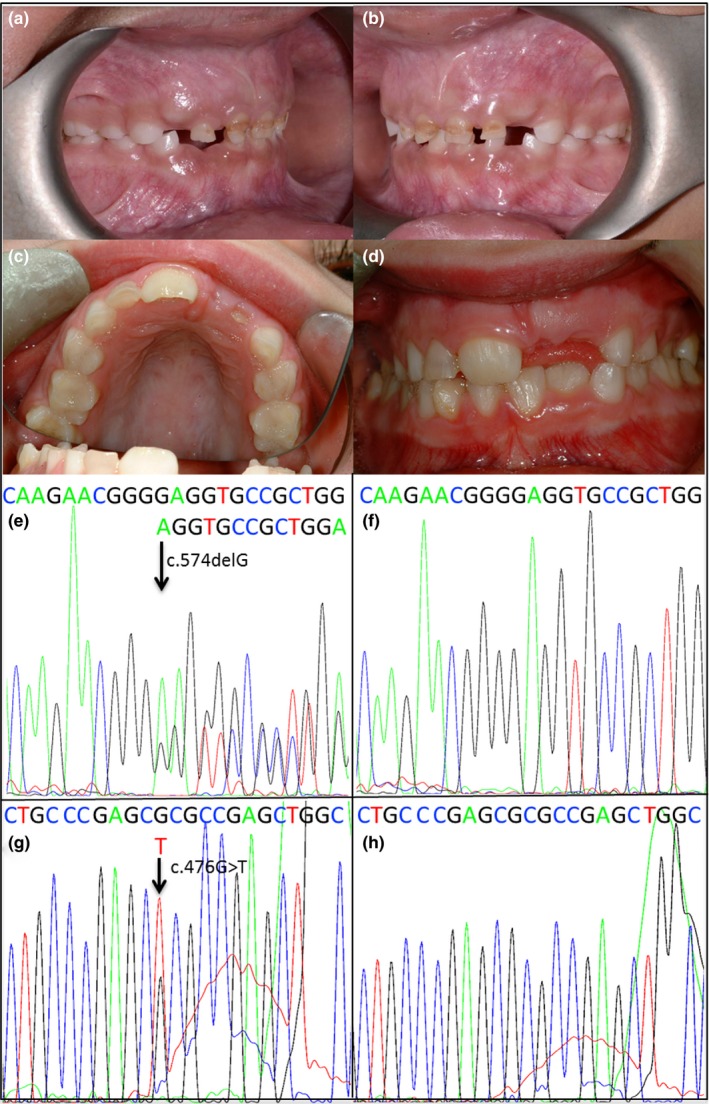
Clinical photographs and DNA sequencing electropherograms for families 1 and 2. (a, b) Clinical photographs of family 1 individual V:2. Note the carious lesions and thin, hypoplastic, pitted enamel. (c, d) Clinical photographs of family 2 individual III:1. Note worn, hypoplastic enamel. e‐f: Sanger sequencing electropherograms for family 1; affected individual V2 (e), unaffected individual V:1 (f). (g, h) Sanger sequencing electropherograms for family 2; affected individual II:2 (g) and family 2 individual II:1 (h). Sanger sequencing reads for other members of family 1 are available in Supporting Information Figure S6 [Colour figure can be viewed at wileyonlinelibrary.com]

Indel and single‐nucleotide variants were called in the VCF format using the Haplotype Caller function of the GATK program. Using the VCFhacks package (freely available at https://github.com/gantzgraf/vcfhacks), variants present in NCBI's dbSNP147 or the Exome Aggregation Consortium database (ExAC; v0.3.1) (Lek et al., 2016) with a minor allele frequency (MAF) ≥0.1% were excluded. Genes known to be associated with autosomal dominant AI were then identified from the variants list and segregated with Sanger sequencing for all family members for which DNA was available. Sanger sequencing was performed using the BigDye Terminator v3.1 kit (Life Technologies, Carlsbad, CA, USA) according to manufacturer's instructions and resolved on an ABI3130xl sequencer (Life Technologies). Results were analysed using SeqScape v2.5 (Life Technologies) (Figure 2).

For families 2 and 3, clinical exome libraries were generated from genomic DNA from individual II:2 (family 2) and individual II:1 (family 3) (marked with arrows on the pedigrees, Figure 1). Targeted clinical exome sequencing was performed on three micrograms of genomic DNA using the Agilent SureSelect Clinical Research Exome V1 (Agilent Technologies), according to the manufacturer's instructions. Captured libraries were pooled and sequencing performed on an Illumina HiSeq2500 sequencer (Illumina) using rapid mode. The resulting fastq files were aligned to the human reference genome (GRCh37) using BWA and reads processed using SAMtools, Picard and the Genome Analysis Toolkit. Variants within the captured regions were called and annotated using AlamutHT (Interactive Biosoftware, Rouen, France) and further filtered to identify variants within genes known to be associated with amelogenesis imperfecta. For family 2, Sanger sequencing to confirm segregation was carried out as above (Figure 2).

Primer sequences for Sanger sequencing of both point mutations can be found in Supporting Information Table S1. Gene lists used for filtering for both families are available in Supporting Information Table S2.

Different methods were employed for each family because families 2 and 3 were part of a pilot study for NHS screening of known genes for AI and screened patients from the newly implemented NHS screening service, respectively (Holland, 2017). Family 1 were recruited as part of ongoing research into the genetic basis of AI, based at the Faculty of Medicine and Health, University of Leeds.

### 2.3 Hair phenotyping

**Figure 3:**
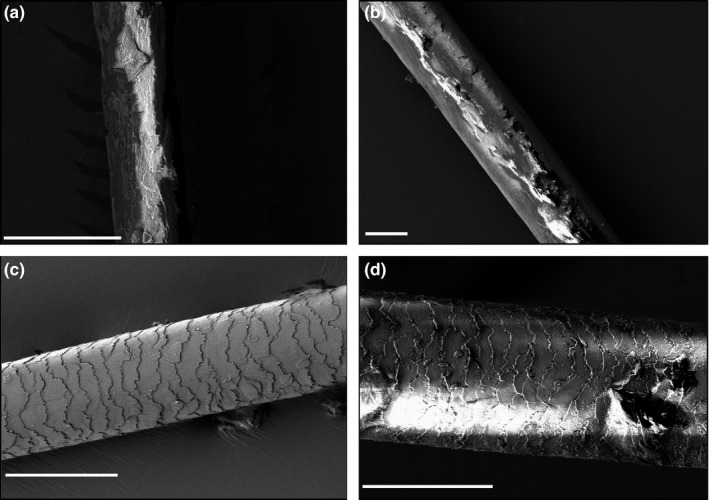
Samples of hair from family 1, imaged longitudinally using Hitachi S‐3400N scanning electron microscope to investigate differences in hair structure between affected and unaffected individuals. a = V:2 (affected), b = IV:4 (affected), c = V:1 (unaffected) normal area, d = V:1 (unaffected) irregular area. Scale bar = 50 µm

Samples of hair from family 1 were sectioned perpendicular to the shaft using a scalpel and mounted on aluminium stubs. Samples were held in place with conductive Acheson Silver DAG 1,415 M paint (Agar Scientific, Elektron Technology, Stansted, UK) and sputter coated with gold using an auto sputter coater (Agar Scientific). The hair was mounted flat (along the longitudinal axis of the shaft) or upright (on the cut end). The hair was imaged using a Hitachi S‐3400N scanning electron microscope (Hitachi, Tokyo, Japan), fitted with a 123 eV Nano XFlash® Detector 5010 (Bruker, Billerica, MA, USA) and operated at an accelerating voltage of 5 kV using back‐scatter electron detection. One strand of hair from each of the three available hair samples was analysed (Figure 3).

### 2.4 iTasser

Predicted protein outcome for the missense variant found in family 2 was assessed using the online protein structure and predictions resource iTasser (Yang & Zhang, 2015). Wild‐type and mutant residues of DLX3 were input and the predicted outcomes for active sites and the secondary and tertiary structures of the proteins compared.

## 3 RESULTS

One mixed race (family 1—Afro‐Caribbean and Caucasian) British family and one Caucasian British family (family 2), both with autosomal dominantly inherited hypoplastic AI, and one British Caucasian individual affected with hypoplastic AI but with no family history of AI (family 3) (Figure 1), were recruited to this study as part of a larger cohort of AI families. Families 1 and 2 did not display any clinically obvious co‐segregating health problems, but a kinky, curly hair phenotype was reported in family 1. The affected member of family 3 had AI, delayed tooth eruption, taurodontism, kinky/curly hair phenotype in early childhood and seizures.

Clinical photographs of affected individual V:2 from family 1 revealed thin, worn, enamel (Figure 2). The family reported curly, unmanageable hair which normalized from adolescence. Taurodontism was reported in the clinical records of affected individuals and can be seen in the dental panoramic tomograph (DPT) of individual IV:4 (Supporting Information Figure S1). Clinically evident bony changes (e.g., mandibular prognathism, Robinson & Miller, 1966) were not reported.

Bitewing radiographs (Supporting Information Figure S1) and clinical photographs (Figure 2) for individual III:1 from family 2 evidence thin, hypoplastic enamel and mild taurodontism of the first permanent molars and premature tooth wear. Family 2 reported no abnormal hair phenotype. Similar to family 1, no abnormal bone phenotype was reported for family 2, although no specialist radiological assessment was undertaken and the alveolar bone on intra‐oral dental radiographs appeared normal. DNA extracted from the buccal swab of individual III:1 was of insufficient quantity for analysis.

Clinical photographs and radiographs from individual II:1, family 3 (Supporting Information Figures S1 and S2) reveal hypoplastic enamel and marked taurodontism of both the primary and secondary dentition, with tooth surface loss and caries. No hypodontia was reported. Delayed eruption of the upper lateral permanent incisor teeth was a feature, along with talon cusps evident on radiography on these teeth, but no bony changes were identified in the gnathic bones on dental radiographs, nor were they reported by family members. One tooth in the permanent dentition had internal resorption and subsequent fracture. A curly/kinky hair phenotype was identified in II:1 in early childhood, but this normalized with age and the individual's parents were not aware of a kinky/curly hair phenotype in their childhoods.

For all of our families, there was no clinical justification for formal bone assessment involving ionizing radiation to confirm or exclude a phenotype consistent with TDO.

DNA from individual IV:1 (family 1) was screened by WES. Individual II:2 (family 2) and individual II:1 (family 3) were screened by targeted clinical exome sequencing. The percentage of bases covered at least 15 times for the regions targeted by each capture type was 96.8%, 99.5% and 99.0% for individuals IV:1 (family 1), individual II:2 (family 2) and individual II:1 (family 3), respectively. Since AI in families 1 and 2 displayed a clear dominant inheritance pattern, including male‐to‐male transmission, only rare (MAF <0.1%), heterozygous, autosomal variants were selected. Variants in genes known to be associated with an AI phenotype were prioritized for investigation. For family 1, this highlighted a one‐base pair deletion in *DLX3*, c.574delG (NM_005220.2), deleting a base in the third and final exon. For family 2, a substitution was identified, c.476G>T; p.R159L (NM_005220.2) affecting a base in the second exon of the *DLX3* transcript. Sanger sequencing confirmed segregation of these variants with disease in all available family members (Figure 2). Supporting Information Figure S3 shows the Integrated Genomics Viewer reads for family 1 to confirm the presence of a 1‐bp deletion in clonal sequencing.

The c.574delG variant identified in family 1 is predicted to result in a frameshift p.(E192Rfs*66) (NP_005211.1). However, the transcript produced is likely to escape nonsense‐mediated decay because it is in the final exon (Isken & Maquat, 2008). The variant transcript is predicted to encode a protein of 256 amino acids (compared to the 287 amino acid wild‐type protein) with 65 incorrect amino acids incorporated at the C‐terminus.

The variant from family 2 incorporates a hydrophobic leucine residue instead of a positively charged arginine residue. When the p.R159L‐mutated DLX3 protein identified in family 2 was input into iTasser, the resultant protein structure was significantly different from that predicted for wild‐type DLX3. Protein p.159L is predicted to have a more tightly packed arrangement, with additional helical regions (Supporting Information Figure S4) and loss of predicted methionine and propanoic acid ligand‐binding sites.

The c.574delG and c.476G>T variants are absent from databases of variation, including NCBI's dbSNP147, the Exome Variant Server, ExAC (v.0.3.1) and gnomAD. The c.574delG and c.476G>T variants were assigned a phred‐scaled CADD (v1.3) score of 34 and 35, respectively, indicating that they are amongst the top 0.04% most predicted deleterious variants analysed by CADD (Kircher et al., 2014). For the missense variant, c.476G>T, the HumVar‐trained PolyPhen‐2 model gave a pathogenicity score of 1.0, predicting it to be damaging, and likely pathogenic (Adzhubei et al., 2010). MutationTaster predicted the c.476G>T variant to be disease causing, with a probability value of 0.999, again suggesting the missense variant is likely pathogenic (Schwarz, Cooper, Schuelke, & Seelow, 2014). The mutations both affect residues in evolutionarily conserved regions of the DLX3 protein sequence (Supporting Information Figure S5), with only the African elephant displaying variation at a position equivalent to p.159 with an alanine residue instead of an arginine residue.

**Figure 4:**
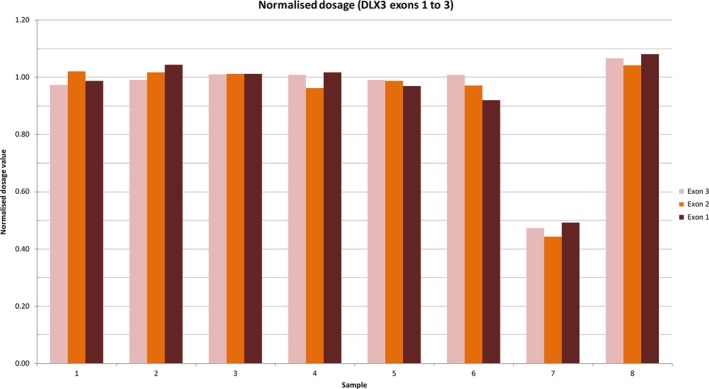
Comparative read‐depth analysis of next‐generation sequence at the *DLX3* locus in family 3. Read depth was corrected for sample‐to‐sample variations by dividing the total number of reads for each exon in each sample in an NGS lane by the average global read‐depth for the sample. That value was then compared to the median value for the dataset. Average read‐depth across the *DLX3* gene for the 8 samples was 1,246×. The normalized dosage ratios for exons 1 to 3, respectively, for the patient sample were 0.49, 0.44, 0.47, implying heterozygous deletion of the entire *DLX3* gene [Colour figure can be viewed at wileyonlinelibrary.com]

For individual II:1, family 3, a heterozygous deletion of the entire coding region of *DLX3* was detected by comparative NGS read‐depth analysis (Green et al., 2017). After correcting for sample‐to‐sample variation in each of the samples in the same sequencing lane (*n* = 8), read depths across *DLX3* exons for individual II:1 were compared to the median value for the dataset, effectively using the other seven sequenced samples in the lane as a control data set for the patient sample. Average read‐depth across the *DLX3* gene for the eight individuals was 1,246×, while the normalized dosage ratios for exons 1 to 3, respectively, for our patient sample were 0.49, 0.44, 0.47 (Figure 4), corresponding to a heterozygous deletion of the entire gene. This was subsequently confirmed by an NHS clinical laboratory using real‐time PCR with primers mapping within the deleted region (data not shown).

Due to the discontiguous nature of clinical exome sequencing, the exact breakpoints are unknown. The nearest adjacent loci showing normal dosage in the patient are at positions chr17:45,924,645 and chr17:48,263,029 on human genome assembly GRCh37/hg19, giving a maximum size of approximately 2.3 Mb, though it is likely that the deletion is smaller than this. Deletions including *DLX3* were not present in ExAC, but a larger deletion also encompassing the genes flanking *DLX3* was identified in one individual in the Database of Genomic Variants (DGV; nsv833474 (MacDonald, Ziman, Yuen, Feuk, & Scherer, 2013)). A familial case of TDO with osteogenesis imperfecta and intellectual disability has also been reported, associated with a heterozygous deletion of 3.4 Mb, including *DLX3* (Harbuz et al., 2013).

The variants from families 1 and 2 were submitted to ClinVar (SCV000583455 and SCV000583456) and were entered in the Leeds AI Genetics LOVD (http://dna2.leeds.ac.uk/LOVD/).

Hair samples were obtained from three individuals in family 1: IV:4, V:2 and V:1 (two affected and one unaffected). These were removed directly from the scalp. Analysis revealed a marked difference between the affected and unaffected individuals (Figure 3). No cuticle was evident for the affected individuals for the hair that was analysed. This gave the hair shaft a woody, brittle appearance upon imaging by SEM. In comparison, the unaffected family member had a largely normal hair shaft, comprised of the typical lamellar cuticle scales, with only occasional areas of irregularity in their arrangement. All of the samples were approximately round in cross section.

## 4 DISCUSSION

**Figure 5:**
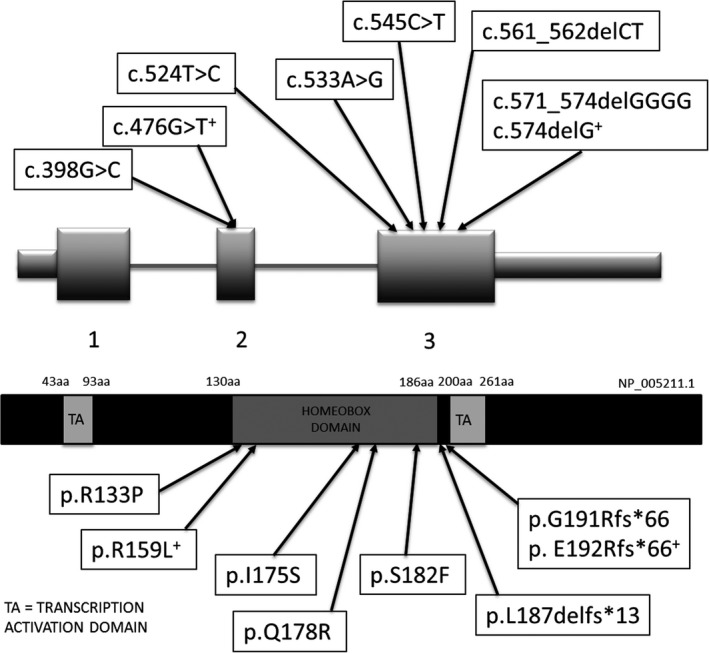
Diagrammatic representation of the position of reported mutations in *DLX3* and the corresponding predicted changes in DLX3 (not to scale). Variants marked with ^+^ are those reported in this paper. c.398G>C, p.(R133P) (Nieminen et al., 2011), c.524T>C, p.(I175S) (Mayer et al., 2010), c.533A>G, p.(Q178R) (Li et al., 2015), c.545C>T, p.(S182F) (Nieminen et al., 2011), c.561_562delCT, p.(L187delfs*13) (Dong et al., 2005), c.571_574delGGGG, p.(G191Rfs*66) (Price et al., 1998)

Here, we report the first families segregating the *DLX3* c.574delG, p.(E192Rfs*66) or c.476G>T, p.(R159L) variants and give a second report of a complete heterozygous deletion of *DLX3*, found through whole‐exome sequencing (family 1) or targeted clinical exome sequencing (families 2 and 3). Figure 5 plots the positions of these pathogenic variants in the *DLX3* gene and the corresponding predicted changes in DLX3 protein. Hypoplastic AI and taurodontism were phenotypic features in all families. Brittle, wavy hair changes were identified in affected members of family 1, and a kinky/curly hair phenotype in childhood was identified in the affected individual in family 3. No abnormal hair phenotype was identified in family 2. No bone, nail or skin changes were reported in any of the families. Individual II:1 from family 3 was also reported to have seizures—a previously unreported finding for TDO. Without knowing the breakpoints of this deletion, it is difficult to know whether this phenotype is wholly attributable to the *DLX3* deletion, or whether other genes flanking *DLX3* may have also been deleted. DLX3 is known to play a role in craniofacial development and is thought to be involved in the development of the ventral forebrain, which may explain the seizures (Zhu & Bendall, 2006). However, the possibility of involvement of the adjacent genes (e.g., *DLX4* or *ITGA3*) in the resultant phenotype needs to be considered. This mutation in *DLX3*, in contrast to the autosomal dominant mutations seen in families 1 and 2, may have arisen *de novo* or could be the result of inheritance from one parent, with partial penetrance of the phenotype.

The variants discussed in this paper were detected using two different methods; for family 1, whole‐exome sequencing was used, whilst for families 2 and 3 targeted clinical exome sequencing was used. Clinical exome sequencing has the advantages over whole‐exome sequencing of providing higher coverage for targeted areas, a lower cost per sample analysed and easier interpretation. However, whole‐exome sequencing allows the identification of variants in new genes for AI.

*DLX3* is comprised of 3 exons and is located on the long arm of chromosome 17, along with other members of the distal‐less family of genes (*DLX1–6*). It is expressed in the placenta and is considered to have a pivotal role in hard‐tissue formation, possibly explaining why the *DLX3* null genotype is fatal in embryo mice (Hwang, Mehrani, Millar, & Morasso, 2008; Morasso, Grinberg, Robinson, Sargent, & Mahon, 1999). DLX3 is crucial for hair cycling (Hwang et al., 2008), patterning of the embryonic ectoderm (Park & Morasso, 2002), and is implicated in bone formation (Hassan et al., 2004). It is a transcriptional activator, expressed in the differentiated epidermal granular cell layer and in the matrix cells of hair follicles. It has also recently been shown to affect ion transport in amelogenesis through regulation of genes in the solute carrier (*Slc*) family (Duverger, Ohara, Bible, Zah, & Morasso, 2017).

The phenotype of the affected individuals in all three of our families is similar to that seen in TDO, but without clinically obvious bony changes, nail features and dermatitis. Conducting bone scans and radiographs using ionizing radiation to characterize any bone changes could not be justified in the context of the clinical presentations. The bony changes in TDO are considered to be variable and can be difficult to characterize, despite reportedly being present in 65%–80% of patients (Price et al., 1998). It has also been suggested that bony changes progress with age, meaning that while not currently clinically apparent, the bony features may be more noticeable as the younger affected individuals age (Hart et al., 1997; Price et al., 1998). The increase in bone density seen in TDO patients has been hypothesized to be due to decreased osteoclastic bone due to the increased IFN‐γ expression by immune cells, although the pathway is not fully elucidated (Choi et al., 2009). It is currently unclear whether bones outside of the craniofacial area are also affected in TDO; however, Haldeman et al. (2004) demonstrated that bone density was increased at the radius and ulna sites in individuals with TDO at ages less than 30 years but showed no obvious association between 30 and 74 years of age. Bone density of the spine and hip was also elevated in individuals with TDO, even at a young age. One of the more common, and easily identifiable, bony features of TDO is mandibular prognathism. In a study by Nguyen, Phillips, Frazier‐Bower, and Wright (2013), TDO patients were compared to wild‐type controls and no mandibular prognathism was identified. However, a retrusive maxilla, increased mandibular length and increased ramus height were common to most TDO cases, possibly giving the appearance of a prognathic mandible in affected individuals.

DLX3 has three main domains: the N‐ and C‐terminus transactivation domains, and a central homeodomain, encoded by exons 2 and 3. The homeodomain can interact directly with DNA in a sequence‐specific way and regulates the expression of target genes throughout numerous developmental processes (Feledy, Morasso, Jang, & Sargent, 1999). All of the previously reported mutations in *DLX3* (each with an attenuated TDO‐like phenotype) have affected residues within, or adjacent to, the homeodomain (Figure 4), altering the structure of this region and highlighting its importance in the pathogenesis of TDO. The addition of these variants to the existing literature highlights the different presentations associated with *DLX3* variants and demonstrates, for all of our families, a more restricted phenotype than classical TDO.

The literature around TDO states that the hair of affected individuals straightens with age in 54% of affected individuals (Wright et al, 1997), and members of family 1 reported that their kinky/curly hair phenotype noted in childhood straightened with age. However, this changing phenotype does not seem to have been structurally demonstrated with SEM imaging of affected individuals from family 1, as the hair from the affected adult (IV:4) was morphologically similar to that from the affected child (V:2), with a woody, brittle appearance and almost complete lack of cuticle. In the unaffected individual, V:1, occasional, patchy, irregular cuticle formation was identified in a largely normal shaft. We have no information as to whether individual V:1 had undergone chemical treatments (e.g., straightening or colouring), which may have led to damage (Figure 3). The lack of a suitable control and the absence of a “hair history” for members of family 1 makes associating the *DLX3* genotype with the curly/kinky hair phenotype, and the structural changes seen with SEM difficult in this case. The affected individual from family 3 was reported to have a kinky/curly hair in early childhood, but this straightened over time. It is difficult to compare our findings to the paper by Wright et al (1997) as SEM was not conducted on the hair samples in that cohort. It would be advantageous to have hair from family 3 to ascertain any structural changes to the hair shaft which may corroborate an abnormal hair phenotype, rather than relying solely on patient reports.

It is unknown whether delayed eruption of the permanent upper lateral incisor teeth in family 3 reflects a further phenotypic variation in TDO. In a TDO case described by Jain, Kaul, Saha, and Sarkar (2017), there was early eruption of the permanent first molar teeth. Otherwise, the case had classical features of TDO without a family history. The authors suggest the mutation arose sporadically. This case was associated with a 4‐bp deletion, but the exact location is not discussed in the report. Jain et al. (2017) hypothesized that the precocious eruption may be due to increased osteoblastic activity around the erupting tooth. As no bony abnormalities were identified clinically or on dental radiographs in family 3, it is difficult to determine the cause of the delayed eruption for our case. DNA from family 3, to confirm the deletion and to determine the exact breakpoints, was unavailable for analysis making comparisons with the cases reported by MacDonald et al. (2013) and Harbuz et al. (2013) difficult. It is therefore unknown whether the copy number variant identified in the individual from family 3 is the same as those reported in the literature. Without knowing the exact breakpoints for this deletion, pathogenicity is difficult to predict, although disease is likely to result from haploinsufficiency. Without genetic testing of the parents of individual II:I from family 3, we cannot reliably confirm any pattern of inheritance for this variant. Partial penetrance and inheritance of the variant from one of the parents could be causative, or the mutation may have arisen *de novo*.

The mechanism by which *DLX3* variants cause disease has not been fully elucidated, with numerous mechanisms hypothesized (Choi et al., 2008; Dong et al., 2005; Duverger et al., 2008, 2017 ; Feledy et al., 1999). There are conflicting reports suggesting both haploinsufficiency and a dominant gain‐of‐function effect (Duverger et al., 2008; Li & Roberson, 2017; Nieminen et al., 2011). Of the *DLX3* variants previously reported to date, the c.571_574delGGGG variant is the most similar to the variant reported here in family 1. It has been previously shown that the mutant DLX3 protein p.(G191Rfs*66) translated from the c.571_574delGGGG transcript is targeted to the nucleus, but unable to bind directly to DNA without prior dimerization with a wild‐type protein (Duverger et al., 2008). This was an unexpected finding as the frameshift would be predicted to affect residues downstream of the homeodomain. It could be hypothesized that, if produced, the mutant p.(E192Rfs*66) protein may be similarly unable to bind to DNA without a wild‐type protein partner. Indeed, Li and Roberson (2017) found the transcriptional activity resulting from the p.(G191Rfs*66) mutant protein was comparable to the wild‐type protein, potentially suggesting a dominant‐negative effect where the mutant protein interferes with the normal binding process of the wild‐type protein produced from the wild‐type allele. However, the findings of Choi et al. (2008) suggest that the p.(G191Rfs*66) protein was able to bind to DNA recognition sequences and potentially causes a gain of function due to the novel 66‐amino acid C‐terminal end, resulting in increased osteoblastic activity. Haploinsufficiency has also been discussed by Nieminen et al. (2011) as a cause for the phenotypic features seen in individuals with the c.571_574delGGGG variant, suggesting the syndromic features of TDO are simply a result of too little wild‐type DLX3 and, as such, a reduction in DLX3 activity. The discovery of a heterozygous deletion of *DLX3* in family 3 supports the haploinsufficiency mechanism of disease over the dominant‐negative mechanism in this family.

The findings of this paper demonstrate the phenotypic spectrum associated with *DLX3* variants. Due to the varying functions of DLX3 and resultant variable phenotype, it is important for the clinician to consider the possibility of syndromic phenotypic features when assessing a patient with any of the features of TDO, to ensure that other potential features such as atopic dermatitis, delayed tooth eruption and pathological bony changes are not overlooked.

This report adds to the variants already reported in genes for AI. Such information improves clinical interpretation and allows for accurate genetic testing and counselling to be offered to patients and their families.

## 5 CONCLUSION

*DLX3* mutations can be associated with classical TDO or attenuated versions of the disease, with varying phenotypes. AI and taurodontism appear to be universal features across all reported cases with an associated clinical impact; nail, hair, bone and skin changes are variably reported.

## CONFLICT OF INTEREST

No conflict of interests declared.

## AUTHOR CONTRIBUTION

LLEW and CELS jointly prepared the manuscript and figures and the literature search. CELS analysed the WES data for family 1. LLEW did the Sanger segregation for families 1 and 2. AP and CJB provided clinical information, photographs and radiographs for family 1 and recruited the family. EAO and LRB provided clinical information, photographs and radiographs for families 2 and 3 and recruited the families. RM, RC, TL, JAP and IB provided genetic data for family 3 and analysis. CFI and AJM are senior authors who founded the amelogenesis genetics research in Leeds and provided guidance on and advice for the research. All authors contributed and reviewed the manuscript.
