# Supplementary figures and images for: Novel DLX3 variants in amelogenesis imperfecta with attenuated tricho‐dento‐osseous syndrome
Source: Oral Dis. 2018 Sep 9;25(1):182–91. doi: 10.1111/odi.12955 (PMC6334507; doi:10.1111/odi.12955)

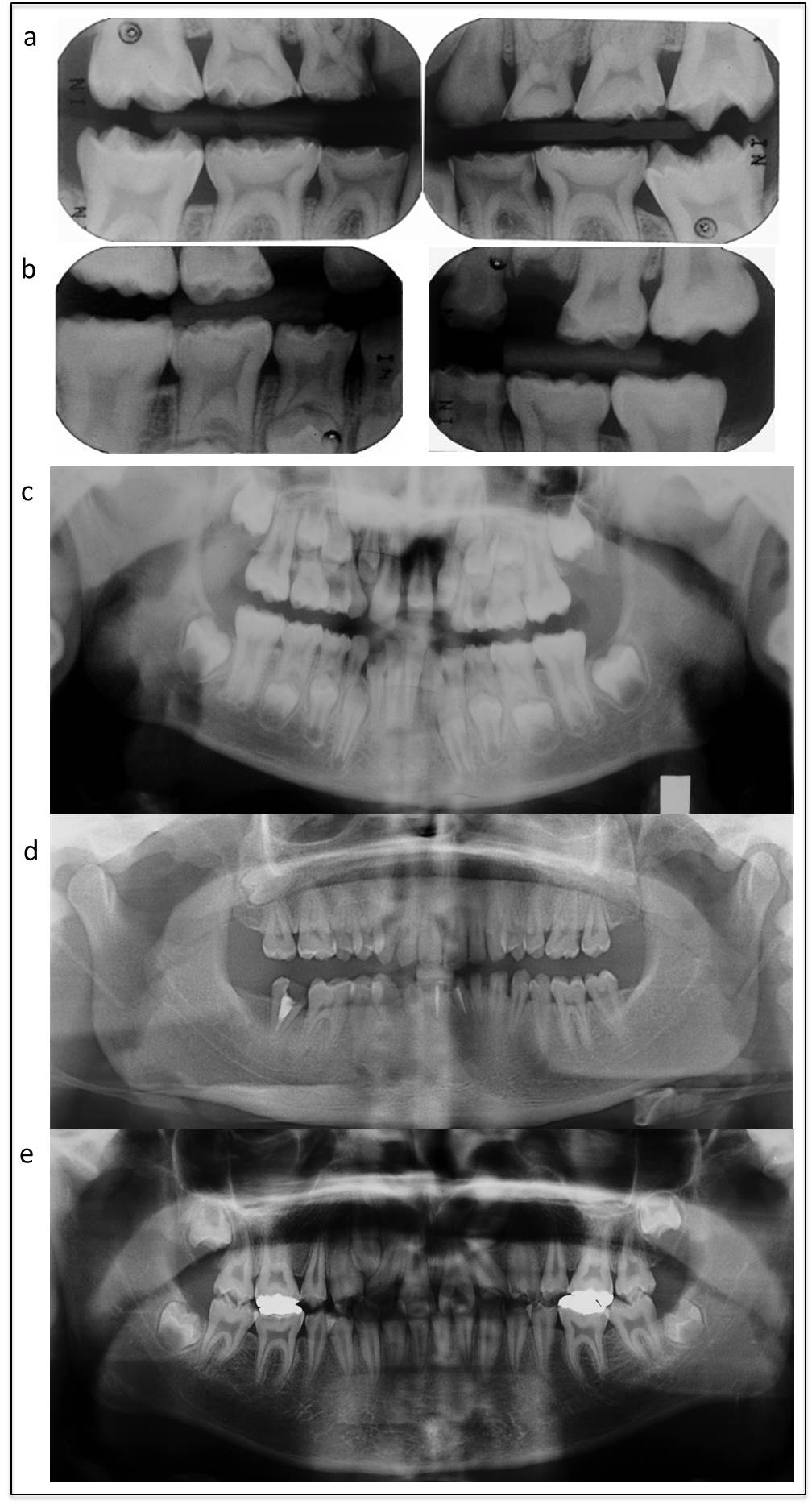

Supplement: Supplementary file 1 [file ODI-25-182-s001.jpg]

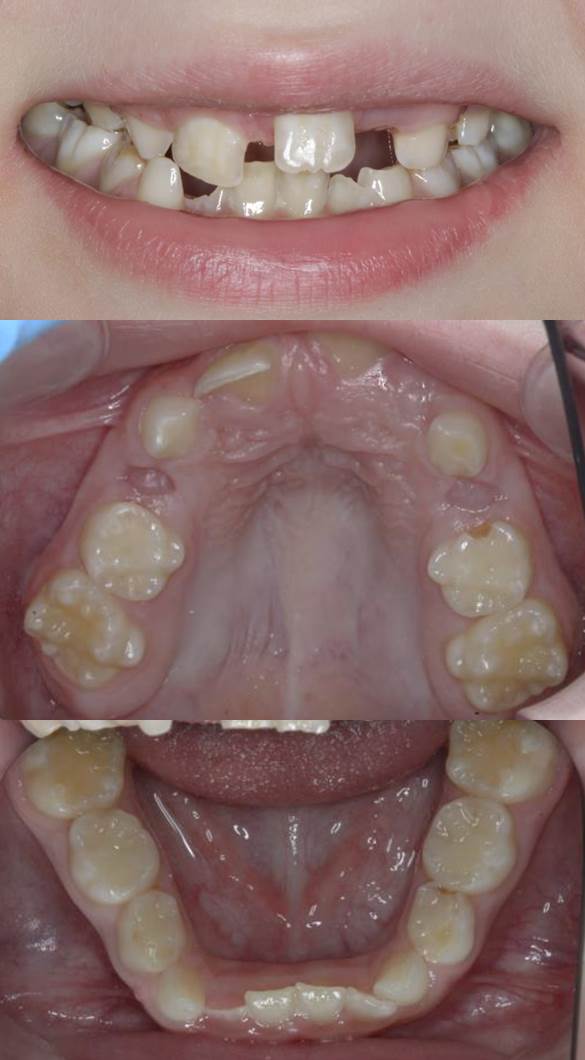

Supplement: Supplementary file 2 [file ODI-25-182-s002.jpg]

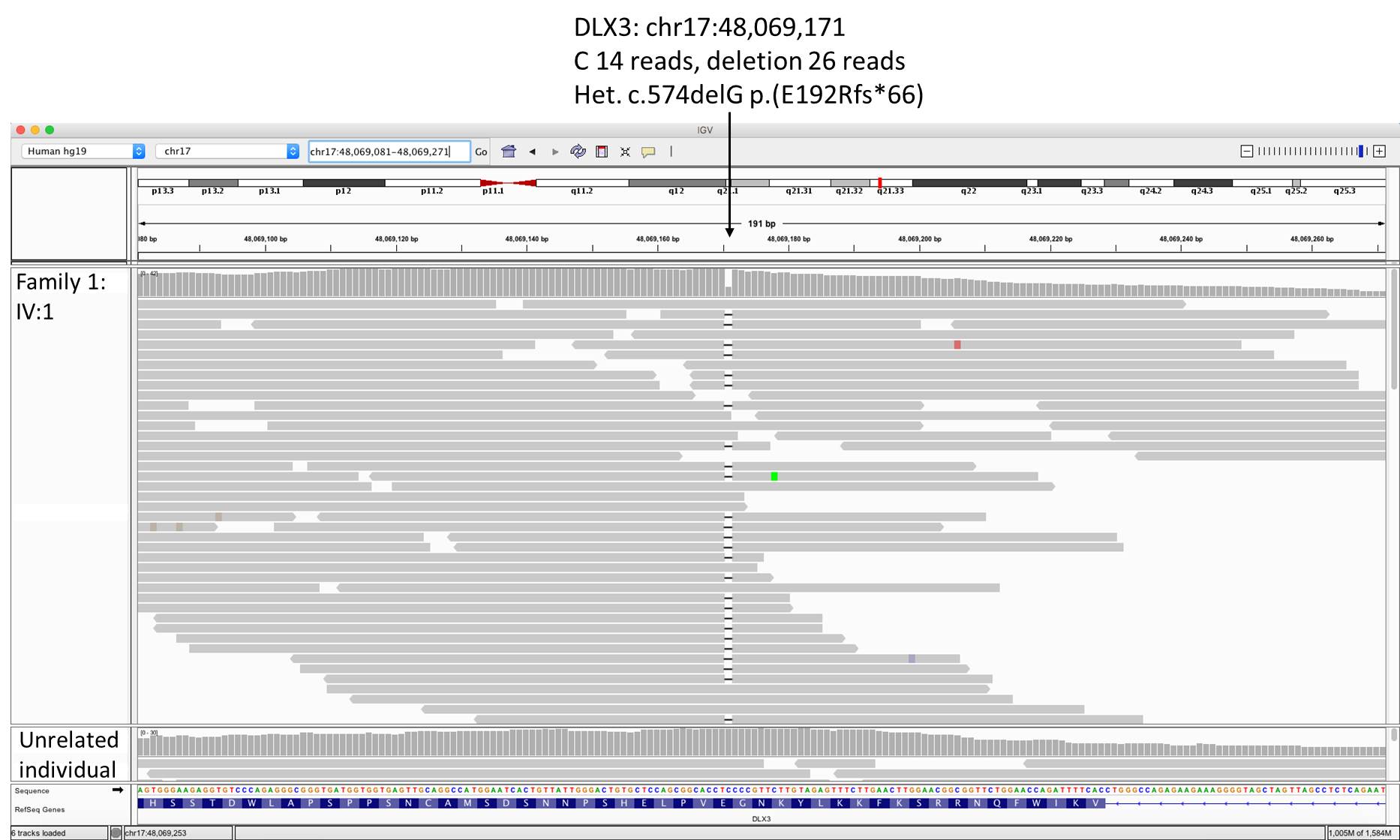

Supplement: Supplementary file 3 [file ODI-25-182-s003.jpg]

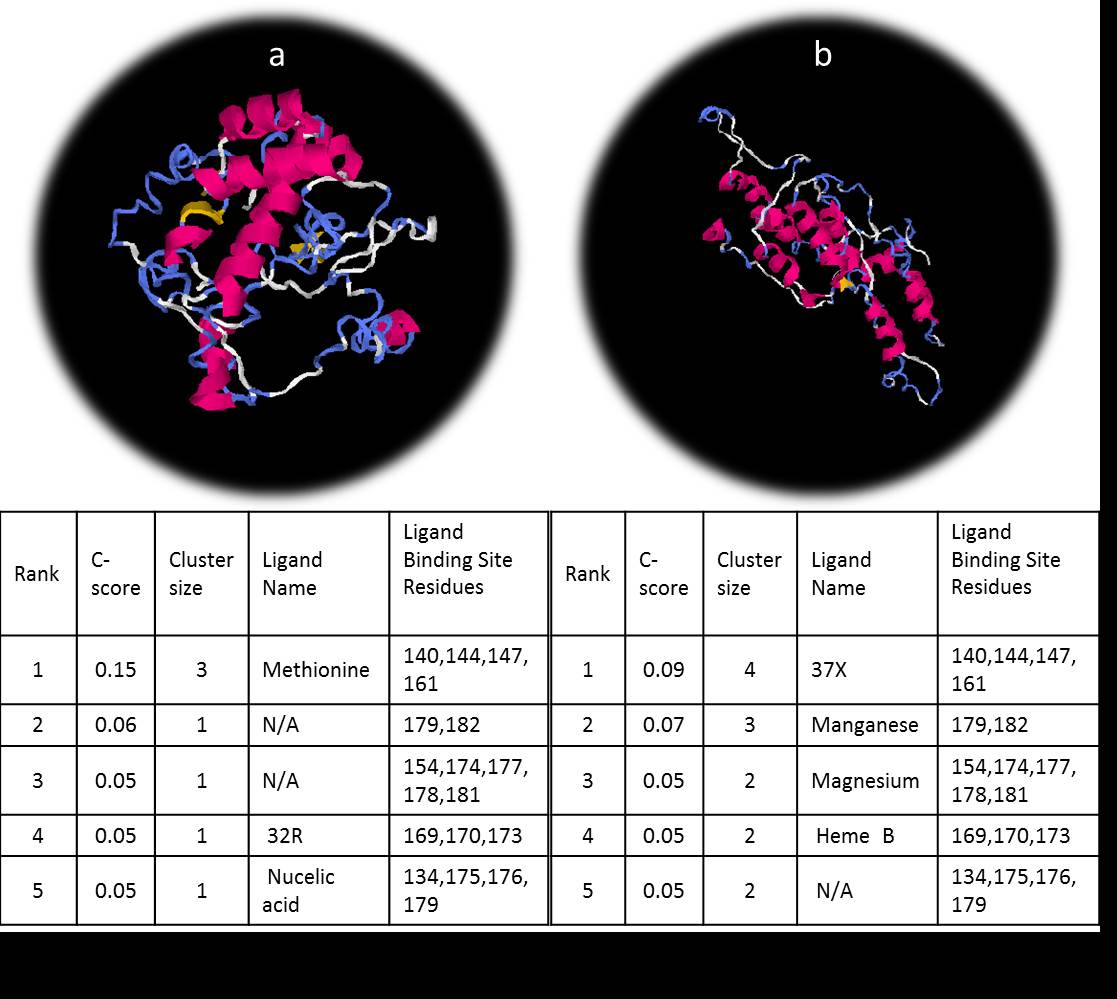

Supplement: Supplementary file 4 [file ODI-25-182-s004.jpg]

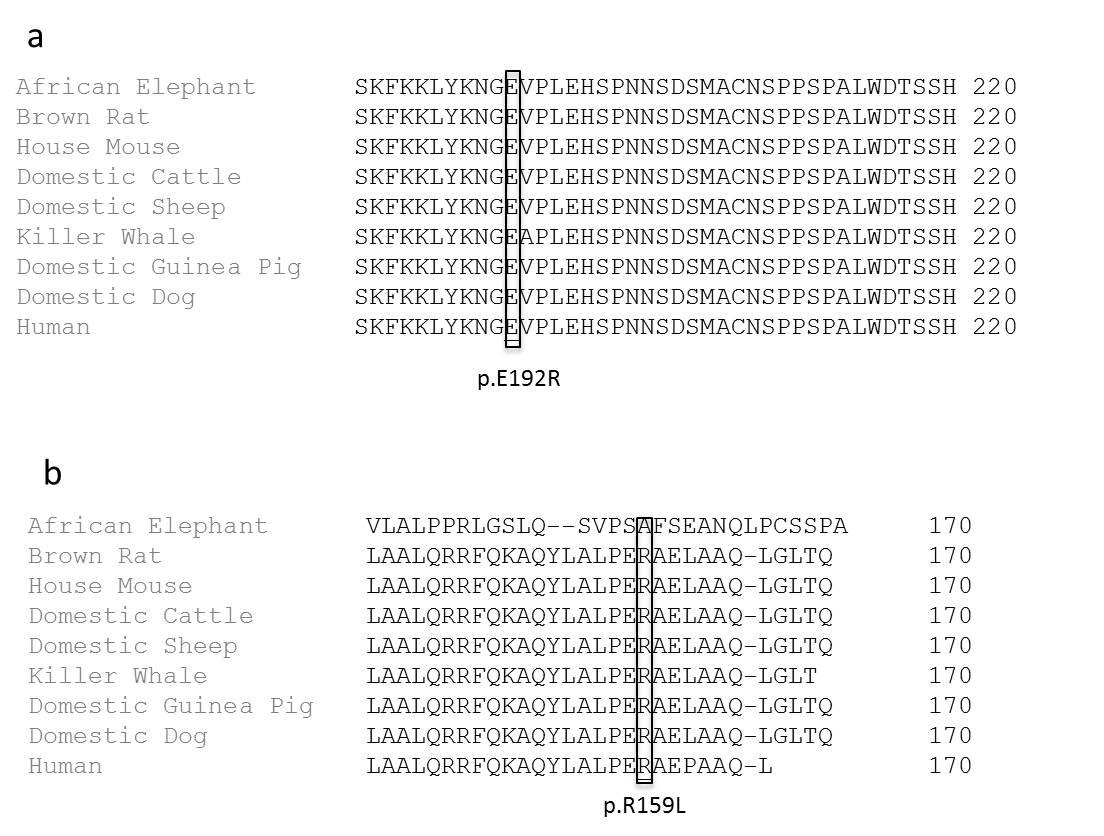

Supplement: Supplementary file 5 [file ODI-25-182-s005.jpg]

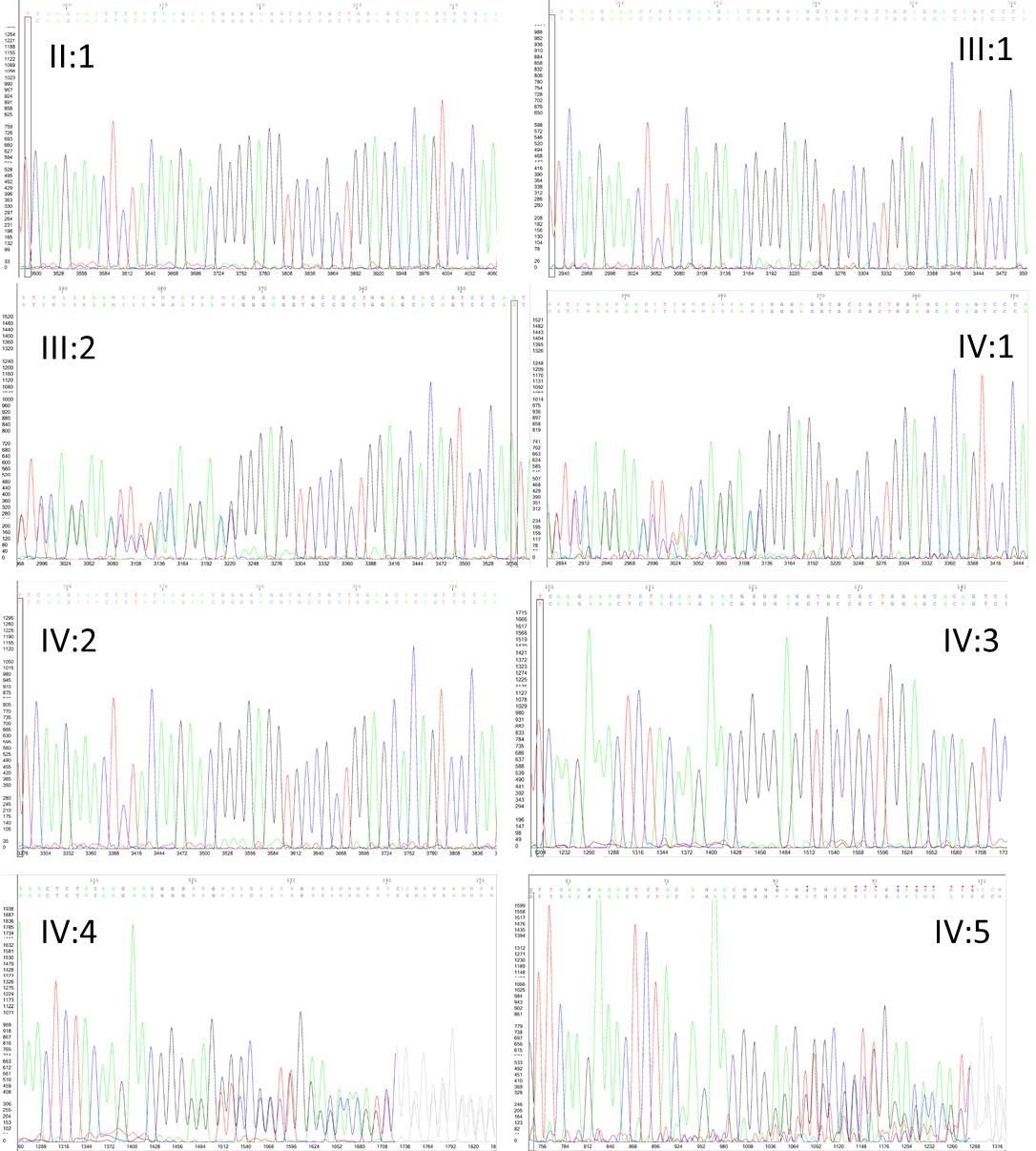

Supplement: Supplementary file 6 [file ODI-25-182-s006.jpg]

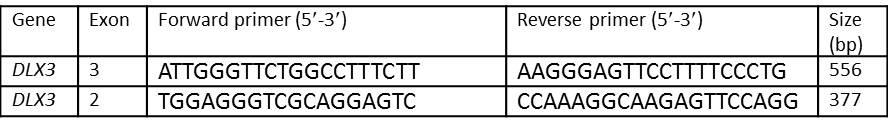

Supplement: Supplementary file 7 [file ODI-25-182-s007.jpg]

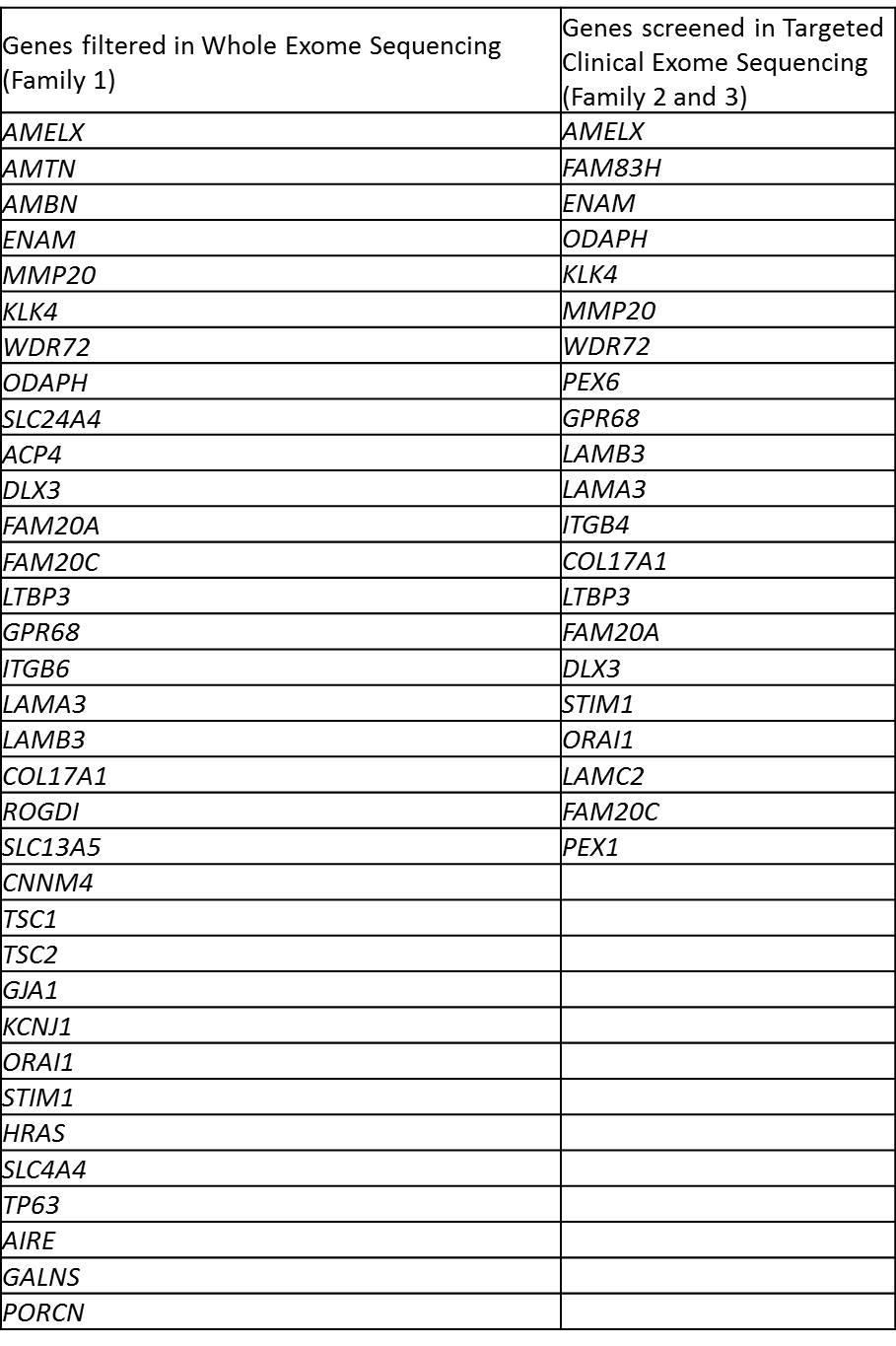

Supplement: Supplementary file 8 [file ODI-25-182-s008.jpg]
